# Temperature effect on the chemomechanical regulation of substeps within the power stroke of a single Myosin II

**DOI:** 10.1038/srep19506

**Published:** 2016-01-20

**Authors:** Chenling Dong, Bin Chen

**Affiliations:** 1 Department of Engineering Mechanics, Zhejiang University, Hangzhou, P.R. China

## Abstract

Myosin IIs in the skeletal muscle are highly efficient nanoscale machines evolved in nature. Understanding how they function can not only bring insights into various biological processes but also provide guidelines to engineer synthetic nanoscale motors working in the vicinity of thermal noise. Though it was clearly demonstrated that the behavior of a skeletal muscle fiber, or that of a single myosin was strongly affected by the temperature, how exactly the temperature affects the kinetics of a single myosin is not fully understood. By adapting the newly developed transitional state model, which successfully explained the intriguing motor force regulation during skeletal muscle contraction, here we systematically explain how exactly the power stroke of a single myosin proceeds, with the consideration of the chemomechanical regulation of sub-steps within the stroke. The adapted theory is then utilized to investigate the temperature effect on various aspects of the power stroke. Our analysis suggests that, though swing rates, the isometric force, and the maximal stroke size all strongly vary with the temperature, the temperature can have a very small effect on the releasable elastic energy within the power stroke.

Myosin IIs are highly efficient nanoscale machines evolved in nature. Within each chemomechanical cycle, a myosin motor converts the chemical energy released from ATP hydrolysis into mechanical energy to perform work^1^,^2^,^3^. Understanding how they function has long been a subject of research interest, which can not only bring insights into various biological processes, including muscle contraction, cell division, cellular mechanosensing, etc., where myosins play critical roles, but also provide guidelines to engineer synthetic nanoscale motors^4^ that efficiently work in the vicinity of thermal noise. Though it was clearly demonstrated that the behavior of a skeletal muscle fiber^5^,^6^, or that of a single myosin^7^ was strongly affected by the temperature, how exactly the temperature affects the kinetics of a single myosin is not fully understood.

Based on structural analyses, how mechanical force was generated by a myosin within the power stroke was explained with the swinging lever-arm theory^8^. According to this theory, a sub-domain within a myosin head behaves as a lever arm, which can rotate about a fulcrum during the power stroke. In this way, a small change within the myosin head can be amplified into the swing of the lever-arm region of the myosin to generate force^9^,^10^.

As indicated in crystallographic models, the total size of a power stroke can be up to ~10 nm^11^,^12^,^13^,^14^, while the motor strain at the isometric state is only ~2nm^15^,^16^. In understanding force recovery in transient tension test, Huxley and Simmons (1971)^17^ proposed a multi-state model, where a myosin can transit sequentially through three sub-states within the power stroke. Similarly, a five-state model was recently proposed^18^,^19^,^20^, where a myosin can transit among five sub-states within the power stroke.

Though these multi-state models provided important insights into the power stroke, such sub-states within the stroke have not been observed so far^21^. Note that these sub-states within the power stroke are different from those bounded nucleotide states suggested by Lymn and Taylor (1971)^1^ or by Hibberd and Trentham (1986)^22^. For example, in the conventional Lymn and Taylor scheme for the chemo-mechanical cycle of a myosin II^1^, the power stroke took place between State AM.ADP.Pi and State AM, and only State AM.ADP was regarded to exist in between within the power stroke.

Most recently, an alternative model was developed^21^, termed as “transitional state model”, where a transitional state associated with a specific event within the power stroke was introduced. In the model^21^, only AM.ADP was associated with the swing of the lever arm, which was load-dependent. The lever arm was arrested at a transitional state, “AM*ADP”, whenever the motor force was ~6 pN. Under this condition, a stroke size of ~2 nm was necessary to maintain a steady isometric state. The transitional state model^21^ predicted that the power stroke were completed with multiple transitional sub-steps, and successfully explained how motor force was rather precisely regulated during skeletal muscle contraction^21^,^23^,^24^.

Here, by providing a detailed molecular picture for the substeps within the power stroke of a single myosin II, we adapt the transitional state model^21^ to investigate the temperature effect on the chemomechanical regulation of several aspects of the power stroke. Our analysis suggests that the temperature can have a very small effect on the releasable elastic energy within the power stroke when motor detachment is not considered, though it strongly affects swing rates, the isometric force, and the maximal stroke size. This finding suggests that myosins might have evolved to efficiently function at varied temperature, which may be beneficial to lower forms of life, whose body temperature cannot be maintained. This work explains how exactly power stroke proceeds and provides important insights into effects of temperature on the regulation of motor behavior at the level of a single molecule.

## Description of the model

**Figure 1:**
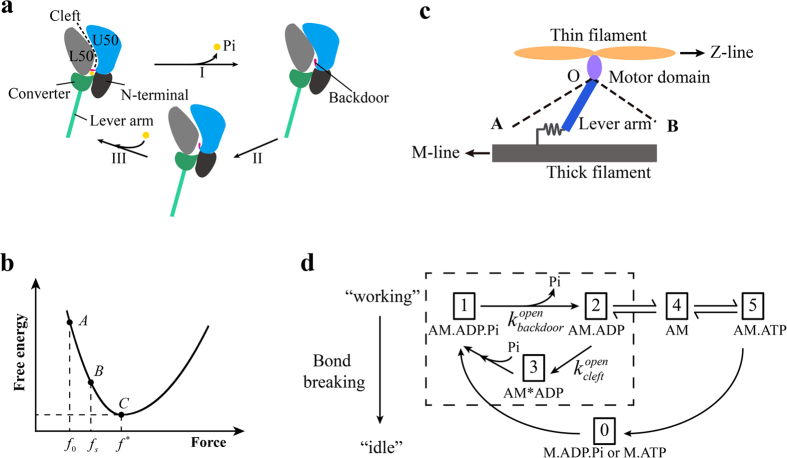
(**a**) Illustration of molecular events within the power stroke. (**b**) Schematics of the variation of free energy against the motor force within the power stroke. Upon swinging from State A towards State C, where the free energy is at the minimum, the lever arm may be arrested at state B. (**c**) Schematics of a single myosin II interacting with the thin filament during the power stroke. OA represents the orientation of the lever arm at the beginning of the power stroke and OB at the end of the power stroke. (**d**) Kinetic scheme of a single myosin II, where states within the power stroke are given in the dashed box. (A, actin; M, myosin motor).

The molecular structure of a myosin II is illustrated in Fig. 1a. The motor domain within each myosin head is made up of several sub-domains that are linked together through flexible connectors^3^,^10^,^25^. Among these connectors, Switch II may serve as the backdoor for the Pi release^9^,^26^, which moves out when the Pi is released^3^,^27^. Between the upper and lower 50-kDa sub-domains is the so-called 50-kDa cleft, which may form the passage for the Pi to be released^28^. This cleft would take a closed form after the Pi release, which blocks the Pi from rebinding^29^.

Similarly to the previous transitional state model^21^, here we hypothesize that the lever arm tends to swing toward a target state, where the motor force would be equal to the isometric force, denoted as 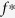. We also hypothesize that motor force can potentially re-open a closed cleft, which would allow a Pi to rebind to the head domain to arrest the lever arm^21^. Based on these two hypotheses, the lever arm may be arrested at an intermediate position of B, for example, at a motor force of f_s_, when it swings from an initial position, A, toward the target state, C, as illustrated in Fig. 1b. Due to structural constraints, the swing of the lever arm should be limited, for example, between OA and OB, as illustrated in Fig. 1c. When the lever arm reaches OB, the stroke size reaches its maximum, 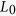, where the power stroke is regarded to be completed.

It should be pointed out that Fig. 1b can be very different from Fig. 3 in Eisenberg *et al.* (1980)^30^, where it was assumed that the attached cross-bridge could exist in two different conformational states with different optimal attachment angles. In Fig. 3 of Eisenberg *et al.* (1980)^30^, the free energy of each cross-bridge state, *G*, was displayed as a function of *x*, a measure of the position of the actin site relative to the cross-bridge of interest. *dG/dx* for an attached state was then regarded as the motor force in that state at *x*.

The molecular picture of substeps within the power stroke is now provided to update the conventional swing lever-arm theory. As illustrated in Fig. 1a, triggered by actin binding upon initial attachment, the backdoor opens, allowing the Pi in the nucleotide pocket to escape through the open cleft^9^,^26^. Once the Pi is released, the cleft closes^29^ and the lever arm swings^31^,^32^, which would generate force on the motor. The lever arm tends to swing toward a target state where the motor force would be 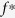. Meanwhile, the motor force can re-open the closed cleft, upon which a Pi enters the nucleotide-binding pocket via the open cleft and the backdoor is closed. The swing of the lever arm would then be arrested with the closed backdoor and the open cleft. Triggered by actin binding, the backdoor can be open again. In this way, the lever arm can swing and be arrested for multiple times and there are multiple transitional substeps within the power stroke, which are chemomechanically regulated.

## Mathematical formulation of the model

We then formulate the adapted transitional state model. In the formulation, the active behavior of the motor is represented with a rigid lever arm that can actively swing around a fulcrum, whereas the passive behavior of the motor is described with a linear spring with a spring constant, 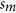
21, as illustrated in Fig. 1c. As marked out in the dashed box in Fig. 1d, there exist three states within the power stroke, including AM.ADP.Pi, AM*ADP, and AM.ADP. The power stroke initiates as an idle motor binds to the actin (AM.ADP.Pi) and releases its Pi. At the state of AM*ADP, both the cleft and the backdoor are open, allowing a Pi to rebind. At the state of AM.ADP, the backdoor is open, but the cleft is closed so that the rebinding of Pi through the cleft is prohibited. Once the power stroke is completed, ADP is released and the motor transits from AM.ADP to AM. By capturing an ATP (AM.ATP), the motor would quickly detach from the actin (M.ATP). A myosin with hydrolyzed ATP (M.ADP.Pi) can rebind to the actin (AM.ADP.Pi) and proceed to the next cycle. Note that a bounded myosin can also detach from the actin directly through bond breaking^21^.

It should be emphasized that we only focus on the kinetics of myosin power stroke shown in the dashed box in Fig. 1d. At the state of AM.ADP.Pi, the initially closed backdoor will open with a rate of 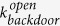, which is taken as a constant for simplicity. When the backdoor is open, the Pi is regarded to be immediately released and the cleft immediately closes. Once the Pi is released, the myosin is at the state of “AM.ADP” with a closed cleft. Denote the initial motor force as 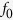, which generally deviates from 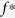. The lever arm then tends to swing. The swing direction and rate are supposed to depend on 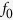
33. When 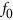 falls below 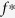, the lever arm mainly swings forward^21^ with a rate given by


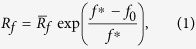


where 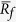 is the forward swinging rate at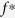. When 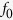 > 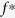, the motor mainly swings backward^21^,^34^ with a rate given by


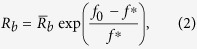


where 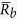 is the backward swinging rate at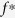. As suggested in Eq. (2), the backward swing rate increases with the stretch size, which is different from the observed relation that the rate of force recovery decreased with the stretch size^17^. This difference can be due to the occurring of motor detachment and rebinding^35^, which may slow down force recovery in experiments.

As illustrated in Fig. 1c, the active swing only occurs between the initial position, OA, and the final position, OB, due to the structural limit. The maximal backward swinging distance would be the net forward swinging distance. If the net forward swing distance is zero, the lever arm will be at OA and cannot actively swing backward so that its backward motion will be completely passive.

The apparent swinging velocity can be related to the swinging rate as


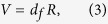


where *R* can be 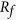 or 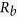, 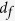 is the strain difference in the motor between the initial position and the target position, i.e., 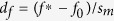. Equation (3) is an approximation, which should be reasonable when the arrested force is not too far from 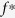.

**Figure 2:**
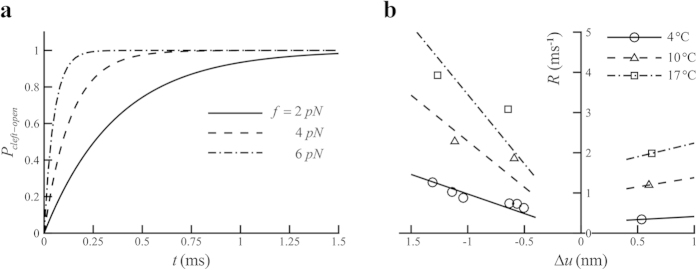
(**a**) Evolution of the open probability of an initial closed cleft with time at different forces with 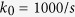 and 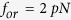. (**b**) Variation of the swinging rate, 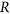, against the strain change in motor, 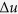. Lines fit to the data points with Eq. (5) or Eq. (6). Data at 4 °C are extracted from ref. 17. Data at 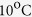 (circles) and 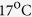 (triangles) are from ref. 11.

During the swinging process, the motor force, *f*, can potentially re-open the closed cleft with the corresponding rate described by the Bell’s law^36^,


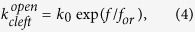


where 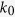 is the opening rate of the cleft without force and 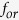 is a reference force, which is just adopted to nondimensionalize 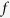. Denote the probability of the cleft at an open state as 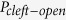, which evolves with time. The one-step master equation for this probability is given as 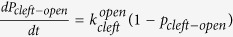. For a cleft that is initially closed, i.e., 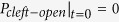, we obtain 
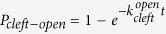
. With Eq. (4), the evolution of 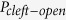 with time at varied forces is plotted in Fig. 2a, showing that the cleft opens at a higher rate at a higher applied force.

With an open cleft, the myosin is at the state of AM*ADP. A Pi is regarded to immediately enter the nucleotide pocket, the backdoor immediately closed, and the swing of the lever arm immediately arrested. The motor is now at the state of AM.ADP.Pi again. The above process can be repeated for multiple times for multiple substeps until the power stroke reaches its maximum. Upon this, the ADP in the nucleotide-binding pocket is released and the myosin reaches the state of  “AM”. Since AM.ADP.Pi, AM.ADP, and AM*ADP can occur multiple times within the power stroke, they are all transitional.

## Analysis of the temperature effect on several aspects of the power stroke

We then investigate the temperature effect on several aspects of the power stroke with the formulated theory. Firstly, our analysis indicates that the temperature has a strong effect on 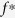, which is given by 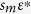, where 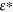 is the isometric strain per motor obtained from muscle transient tests. It was reported that 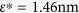 at 2 °C, 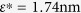 at 5 °C, 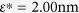 at 10 °C and 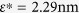 at 17 °C, respectively^7^. With 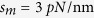
18, we then find that 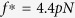 at 2 °C, 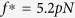 at 5 °C, 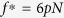 at 10 °C, and 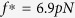 at 17 °C, respectively. With a linear interpolation, we also find 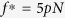 at 4 °C.

Our analysis also indicates that the temperature has a strong effect on the swing rate of the power stroke. Dependence of swing rates of the lever arm on forces within the power stroke can be estimated from r curve in *T*_2_ transient tests^11^, which represents the variation of the dominant rate in force recovery with the length change per half sarcomere. In the estimation, dominant rates in force recovery are regarded as dominant swing rates in the power stroke. With the deduction of the contribution of the elasticity of the filaments in the length change^18^, the variation of dominant swing rates with changes in motor strains at different temperatures is obtained, as plotted in Fig. 2b. On the other hand, the dominant term in the Taylor series in the vicinity of 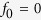 for 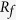 is^21^


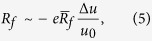


where 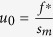 and 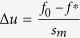. The dominant term in the Taylor series in the vicinity of 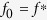 for 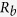 is


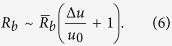


Fitting the data in Fig. 2b for forward swing rate with Eq. (5) yields 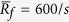 at 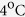, 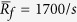 at 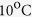, and 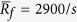 at 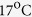, respectively. With the single datum in Fig. 2b for backward swinging rate, we obtain 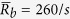 at 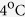, 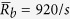 at 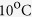, and 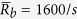 at 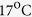, respectively.

**Figure 3:**
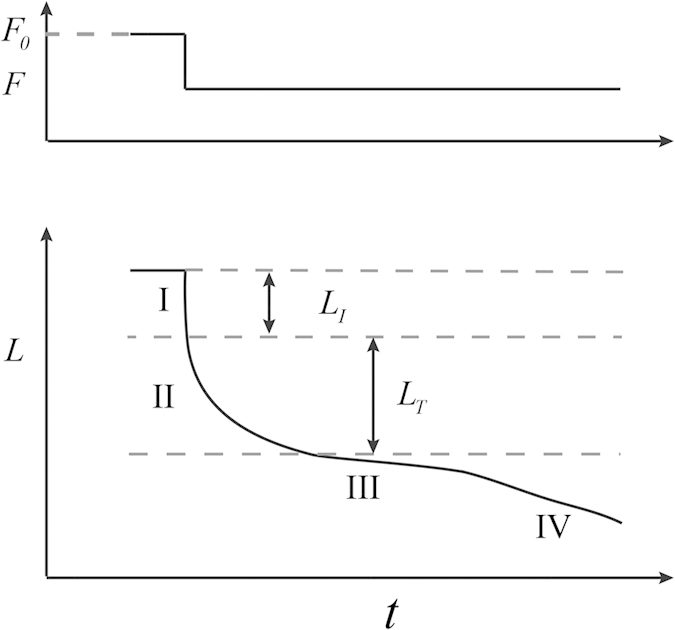
Illustration of 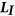, 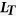 and four phases (lower trace) in velocity transient test after a step change in force, *F*, from the isometric force, 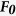 (upper trace).

The temperature also has a strong effect on *L*_0_, which is given by the sum of 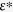 and the maximal size of isotonic power stroke, denoted as 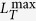, which can be obtained from the transient tests. As illustrated in Fig. 3, there generally exist four phases in the velocity transient tests, where Phase I corresponds to an elastic response with a length change in the half sarcomere of 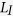 and Phase II is related to execution of the power stroke^37^. 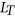, the size of isotonic power stroke, was calculated by subtracting 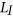 from the length change in the half sarcomere at the end of Phase II. 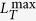 occurred when the filament load was completely relaxed^7^. It was reported that 
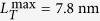
 at 2 °C, 
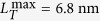
 at 5 °C, 
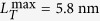
 at 10 °C, and 
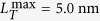
 at 17 °C^7^. With known 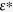 at different temperatures, we find 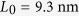 at 2 °C, 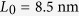 at 5 °C, 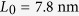 at 10 °C, and, 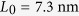 at 17 °C. With a linear interpolation, we also find 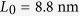 at 4 °C.

**Figure 4:**
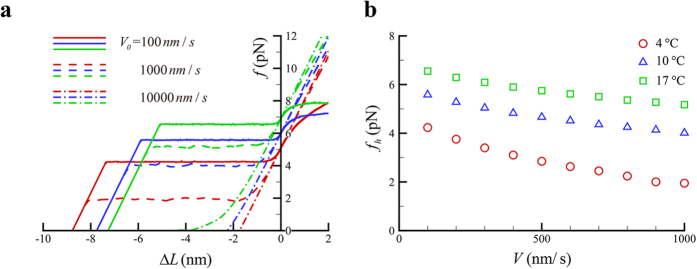
(**a**) Force-length change curves of a bound myosin. Green for 17 °C, blue for 10 °C, and red for 4 °C, respectively. The length change, 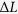, is the sliding displacement of the thin filament relative to the thick filament, which is taken to be negative when the thin filament moves towards M-line and positive when the thin filament moves towards Z-line. (**b**) Variation of 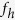 against shortening velocity at different temperature.

With swing rates, 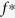, and *L*_0_ obtained at different temperatures, we simulate the effects of temperature on force-length change curves of a single bound myosin with the use of the First Reaction method^38^, which is one type of the Monte Carlo method and valid for non-steady-state kinetics. In this method, a random number, 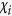, uniformly distributed over (0, 1] is routinely generated for *i*^th^ random event with a reaction rate of 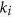 at each time step. The next random event occurs where 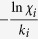 is minimum and the duration for the random event that occurs at the next step is given by the minimum of 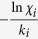. More description of our simulation is provided in Supplemental Information. Some parameters used in the simulation are listed in Table 1. As shown in Fig. 4a, the force-length change curves highly depend on both the temperature and shortening/lengthening velocities. At relatively low shortening velocities, there clearly exist two different phases in force-length change curves at different temperature. At Phase I, the motor force varies little, denoted as 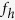, and, at Phase II, the motor force varies almost linearly with the length change at a slope of *s*_*m*_. Calculated 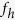 at different temperature is plotted in Fig. 4b, which is generally higher at higher temperature and decreases as the shortening velocity increases.

The area enclosed by a force-length change curve upon shortening and both axes is considered as the released elastic energy, denoted as *W*, which would depend on the temperature and also on shortening velocities. As indicated in Fig. 4a, *W* increases as the shortening velocities decreases until 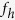 approaches 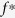 at very low velocities, where *W* reaches the maximum. The maximum of *W* is defined as the releasable elastic energy, denoted as 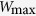, ideally given by


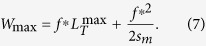


With Eq. (7), 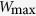 at different temperature is calculated.

**Figure 5:**
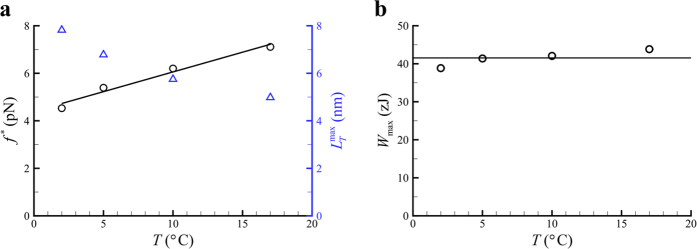
(**a**) Variation of 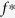, represented with Circles, and 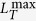 obtained from ref. 7 represented with Triangles, with temperature. (**b**) Variation of 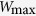 with temperature.

As plotted in Fig. 5a, 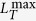 generally decreases as the temperature increases, while 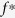 generally increases as the temperature increases. However, as shown in Fig. 5b, we interestingly find that 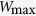 varies little with the temperature and is ~42 zJ. This suggests that the temperature can have a very small effect on the releasable elastic energy within the power stroke, which is ideally defined within the framework of our current theory without considering motor detachment/rebinding.

## Discussion

Though it was suggested that the motor force could be self-regulated in the previous transitional state model^21^, the molecular picture of the substeps within the power stroke was not clear, which is provided in the updated transitional state model in the current work. Within this model, the motor force can re-open the closed cleft to allow a Pi to re-enter the nucleotide pocket within the power stroke. With a Pi in the nucleotide pocket, the swing of the lever arm is arrested. It seems as if the motor is re-primed into the AM.ADP.Pi state before each swing of the lever arm, which leads us to suggest that a portion of mechanical energy may be transformed back into the chemical energy in this process. It should also be noted that the free energy among different AM.ADP.Pi states right before each swing of lever arm may vary.

When the motor force is low, a swing of a large size with a higher rate may occur. When the force on a motor is too high, a Pi rebinding may occur to prevent further swinging of the lever arm. In this way, the filament load can be more or less equally shared among the attached motors so that the function of multiple motors is coordinated. From this point of view, Pis can play critical roles in modulating the coordination among an ensemble of myosin motors.

Coordination between neighboring motors may also be affected by sarcomere lattice geometry or by filament compliance^39^,^40^. It was demonstrated that a mechanical form of cooperativity between neighboring motors might arise from compliant filaments, where motor force resulted in realignment between myosin heads and binding sites along the thin filament. This would lead to additional motor recruitment as force increases^39^. Though Davis and Epstein^41^ suggested that the fiber stiffness increased almost linearly with temperature, Tsaturyan *et al.*^42^ suggested that the fiber compliance didn’t change with temperature. With X-ray diffraction, Linari *et al.*^43^ showed that the higher force generated by skeletal muscle at higher temperature was due to axial tilting of the myosin heads while the fiber compliance didn’t change. Recently, Davis and Epstein^14^ corrected their earlier report^41^ and concluded that the fiber stiffness was independent of the temperature at and above 5 °C.

In this theory, motor force can re-open a closed cleft to allow the re-entry of a Pi. The occupancy of the AM.ADP.Pi state can then increase with the increase of the Pi concentration in the solution. It was found that the bond formed between myosin and actin can break within the power stroke^44^,^45^. Bond breaking may mainly occur at the state of AM.ADP.Pi, since the bond formed at this state can be relatively weaker than that at any other states^46^,^47^. Thus, the number of the attached motors could decrease with the increase of the Pi concentration in the solution^48^.

It should be noted that the releasable energy of a bound myosin is ideally defined by us as the maximal elastic energy it can release within the power stroke without considering motor detachment/rebinding. In experiments, it was revealed that the maximum of 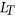 occurred at *F* = 0, with *F* being the fixed filament load during the isotonic shortening^7^. As *F* increased, 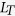 was found to decrease. This can be due to that a bound motor can detach through bond breaking within the power stoke^44^,^45^, which appears to be affected by the motor force. It was further revealed that the difference of 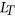 at different temperatures was actually very small in the vicinity of respective isometric loads^7^. For this reason, an energy term, defined as the product of 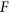 and 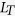, was shown to increase with temperature in experiments^7^.

**Figure 6:**
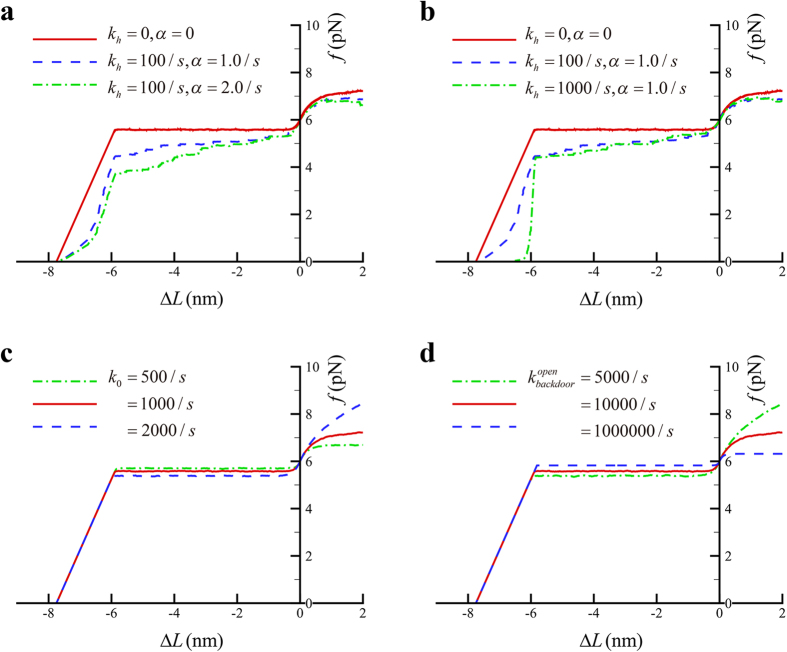
Force-length change curves of a bound myosin II when the rate of catch-bond breaking varies (**a**), the detachment rate through ATP hydrolysis varies (**b**), the rate of cleft opening varies without considering myosin detachment” after (**c**), or the rate of backdoor opening varies without considering myosin detachment (d). Default parameters include 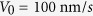, 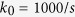, 
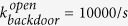
, 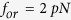, 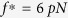, 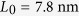, 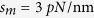, 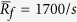, 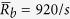.

We have also investigated the case where the motor can detach from the actin. In the consideration, the motor can detach from the actin either through catch-bond breaking^45^ or through ATP hydrolysis cycle by catching an ATP. The catch-bond breaking rate is force-dependent, which is described by^24^,^49^


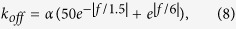


where 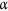 is a rate constant with a unit of 1/s^24^,^50^. The motor can also release its ADP and then quickly detach from actin by catching an ATP. We regard that the motor detaches through ATP hydrolysis cycle at a constant rate, 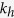, when the size of power stroke reaches its maximum. Following the similar simulation procedure given in the Supplementary Information, we find that the actual size of the stroke or the motor force is affected by the detachment. The calculated force-length change curves in this case are shown in Fig. 6a,b, which suggest that the elastic energy released within the power stroke of a single myosin II *in vivo* can be affected by the detachment.

As shown in Fig. 6c,d, the force-length change curves are also affected by the rate of cleft opening or that of backdoor opening. Increasing the rate of cleft opening tends to decrease the motor force in Fig. 6c. This can be due to that more motors will be arrested at relatively low forces as the rate of cleft opening gets higher. Increasing the rate of backdoor opening tends to increase the motor force in Fig. 6d. This can be due to that the lever arm will swing earlier as the rate of backdoor opening gets higher. Note that the lever arm can only swing after the Pi is released through the open backdoor.

**Figure 7:**
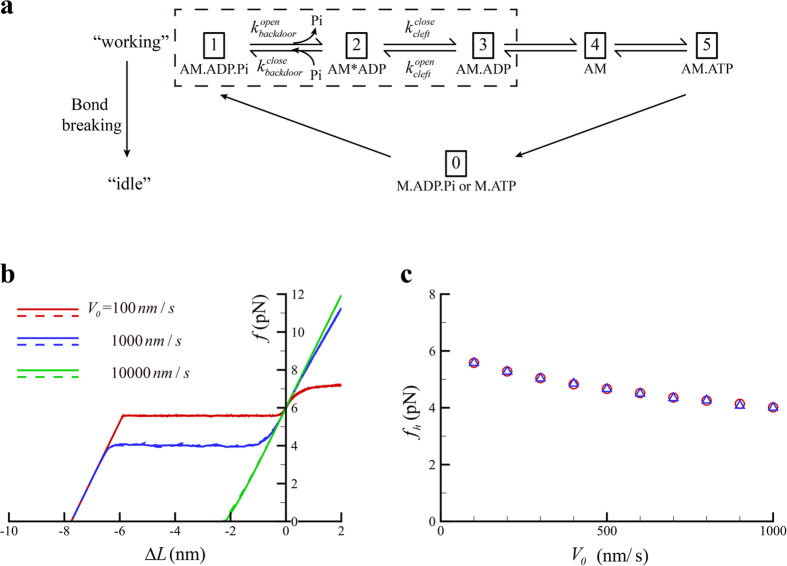
(**a**) Elaborated kinetic scheme of a single myosin II, where states within the power stroke are given in the dashed box. (**b**) Force-length change curves of a bound myosin. Solid curves, which are calculated based on the kinetic scheme marked in the dashed box in (**a**), almost coincide with dashed curves, which are calculated based on the kinetic scheme marked in the dashed box in Fig. 1d. (**c**) Variation of 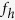 against shortening velocity. Circles, which are calculated based on the kinetic scheme marked in the dashed box in Fig. 1d, almost coincide with Triangles, which are calculated based on the kinetic scheme marked in the dashed box in (**a**). In the simulation, 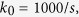 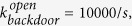 
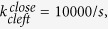
 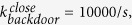 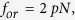 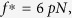 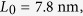 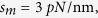 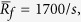 and 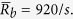

Due to the ATP hydrolysis, some reverse transition rates can be much lower than respective forward transition rates during the power stroke. For this reason, we neglect these reverse state transitions in the analysis above. Now we also consider the case in which these reverse transitions during the power stroke are included into the kinetic scheme, as shown in Fig. 7a. In this new scheme, the motor transits from AM.ADP.Pi to AM*ADP when the backdoor opens and the Pi is released; the motor transits from AM*ADP to AM.ADP when the cleft is closed. In the reverse direction, the motor transits from AM.ADP to AM*ADP when the cleft opens; the motor transits from AM*ADP to AM.ADP.Pi when the Pi rebinds and the backdoor is closed. It should be pointed out that the scheme in Fig. 7a is consistent with that in Fig. 1d when considering that both the cleft and the backdoor close at a very high rate. Let the cleft close at a constant rate, denoted as 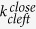, and the backdoor close at a constant rate, denoted as 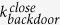. Our simulation indicates that the force-length change curves of a bound myosin and the variation of 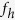 against shortening velocity based on the kinetic scheme marked in the dashed box in Fig. 7a are very similar to those based on the kinetic scheme marked in the dashed box in Fig. 1d, as shown in Fig. 7b,c.

## Conclusion

In conclusion, motor proteins, such as Myosin IIs, are highly efficient nanoscale machines evolved in nature. Here, we try to explain how exactly these soft machines work, which can be of profound significance in both biology and engineering. Our analysis suggests that, though swing rates, the isometric force, and the maximal stroke size all strongly vary with the temperature, the temperature can have a very small effect on the releasable elastic energy within the power stroke.

The releasable elastic energy within the power stroke is calculated to be ~42 zJ. Since the total energy released from the hydrolysis of an ATP molecule can be ~90 zJ under typical cell conditions, the energy efficiency of myosin IIs is ~47%. The independence of such high energy efficiency on temperature may be beneficial to lower forms of life, whose body temperature cannot be maintained.

In the end, we would like to point out that our description of molecular events associated with the power stroke is systematically constructed based on large amount of experimental evidence in the literature, which, however, should be subjected to further experimental trials. It should be also emphasized that the critical assumption in the model is that the working stroke is load dependent, stopping around an isometric force, a condition under which Pi can rebind.

## Additional Information

**How to cite this article**: Dong, C. and Chen, B. Temperature effect on the chemomechanical regulation of substeps within the power stroke of a single Myosin II. *Sci. Rep.* **6**, 19506; doi: 10.1038/srep19506 (2016).

## Supplementary Material

Supplementary Information

## Figures and Tables

**Table 1:** Some default parameters in the simulation.

Parameter	Value	Parameter	Value
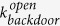	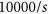	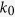	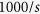
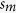	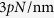 ^18^	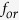	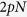
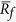	600*/s at* 4 °C 1700*/s at* 10 °C 2900*/s at* 17 °C	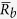	260*/s at* 4 °C 920*/s at* 10 °C 1600*/s at* 17 °C
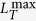	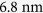 *at* 5 °C^7^ 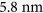 *at* 10 °C^7^ 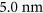 *at* 17 °C^7^	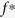	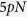 *at* 4 °C 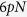 *at* 10 °C 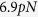 *at* 17 °C
