# Supplementary material for: Temperature effect on the chemomechanical regulation of substeps within the power stroke of a single Myosin II
Source: Sci Rep. 2016 Jan 20;6:19506. doi: 10.1038/srep19506 (PMC4726395; doi:10.1038/srep19506)
Supplement: Supplementary Information [file srep19506-s1.doc]

**Supplementary Information**

Title: Temperature effect on the chemomechanical regulation of substeps within the power stroke of a single Myosin II

Authors: Chenling Dong, Bin Chen

**Force-length change curves of a single bound myosin**

With the mathematical formulation of the adapted transitional state model, the force-length change curve of single bound myosin can be calculated. Consider a single myosin attached to actin, as schematically shown in Fig. 1c. The actin filament, considered as rigid, is then subjected to a step perturbation with a shortening or lengthening velocity, $V_{0}$. Initially, the motor force is $f^{*}$ and the swing of the lever arm is arrested at the state of AM.ADP.Pi. The net distance that the myosin can swing backward is set to be $f^{*}/s_{m}$ at this moment. For example, if $f^{*}\text{=}6pN$, $f^{*}/s_{m}\text{=}2nm$. At some time, the lever arm swings and the motor force evolves as

$f=f_{0}+s_{m}(Vt_{2}-V_{0}t_{1})$ (S1)

where $t_{1}$ is the elapsed time of the step perturbation and $t_{2}$ that of the swing, respectively.

The First Reaction method^1^ is employed to simulate the system. We regard that the motor only swings backward at a lengthening velocity and only swings forward at a shortening velocity. At each time step, there exists only one stochastic event, the opening of the cleft with the corresponding rate given by Eq. (4) or the opening of the backdoor with a constant rate, $k_{backdoor}^{open}$. In determining the time when the next random event occurs, a random number, $\xi$, uniformly distributed over the interval (0,1], is generated^1^. The time interval $\Delta t$ between two successive events is calculated according to

$\int_{0}^{\Delta t} kdt=\ln(1/\xi)$ (S2)

where *k* is $k_{cleft}^{open}$ or $k_{backdoor}^{open}$. The simulation is terminated when the swinging distance is over 2 nm for a lengthening velocity or when the motor force falls below zero for a shortening velocity. The average force-length change curve of a myosin II is obtained by running the simulation for 100 times. Default values for some parameters used in the analysis include $k_{0}=1000/s$, $k_{backdoor}^{open}=10000/s$, $f_{or}=2pN$, and $s_{m}$=3pN/nm^2-4^.

As indicated in Fig. S1(a), calculated force-length change curves of a bound myosin within the power stroke strongly depend on the shortening velocities. At relatively low shortening velocities, for example, 100 nm/s, there clearly exist two different phases. At Phase I, the length change is more or less compensated by the active swing of the lever arm and the motor force varies little, denoted as $f_{h}$. The effects of $f^{*}$ on $f_{h}$ are shown in Fig. S1(b). For different $f^{*}$, there clearly exists $f_{h}$ over a wide range of shortening velocities. As seen in Fig. S1(b), $f_{h}$ generally decreases as the shortening velocity increases. The effects of the cleft open rate on $f_{h}$ are investigated by changing $f_{or}$ in Eq. (4). As shown in Fig. S1(c), decreasing $f_{or}$ would slightly decrease $f_{h}$. A very low $f_{or}$ would result in $f_{h}$ that is significantly deviated from $f^{*}$, suggesting the cooperation of the target force and cleft open rate in regulating the motor force. The effect of $k_{backdoor}^{open}$ on $f^{*}$ is shown in Fig. S1(d), and appears to be weak.

At the end of Phase I, the lever arm reaches the structural limit and cannot swing any more so that the motor force decreases almost linearly with the length change, as seen in Fig. S1(a). Since the motor force is initially at the isometric value, the abscissa intercept of curves in Fig. S1(a) is found to correspond to $L_{0}$ at relatively low shortening velocities.

**Supplemental references**

1. Chen, B. Self-regulation of motor force through chemomechanical coupling in skeletal muscle contraction. *J. Appl. Mech.-Trans. ASME*. *80*, 051013-051013-5 (2013).

2. Fusi, L. *et al*. The non-linear elasticity of the muscle sarcomere and the compliance of myosin motors. *J. Physiol*. *592*, 1109-1118 (2014).

2. Brunello, E. *et al*. The contributions of filaments and cross-bridges to sarcomere compliance in skeletal muscle. J. Physiol. *592*, 3881-3899 (2014).

4. Piazzesi, G. *et al*. Skeletal muscle performance determined by modulation of number of myosin motors rather than motor force or stroke size. Cell *131*, 784-795 (2007).

**Supplemental figures**

Fig. S1 (a) Force-length change curves of a bound myosin II. (b) Variation of $f_{h}$ against shortening velocity at different $f^{*}$. (c) Variation of $f_{h}$ against sliding velocity at different $f_{or}$. (d) Variation of $f_{h}$ against sliding velocity at different $k_{backdoor}^{open}$. In the simulation, $k_{0}=1000/s$, $k_{backdoor}^{open}=10000/s$, $f_{or}=2pN$, $f^{*}=6 pN$, $L_{0}\text{=7.8} \text{nm}$, $s_{m}=3pN/nm$, $\bar{R}_{f}=1700/s$, and $\bar{R}_{b}=920/s$.


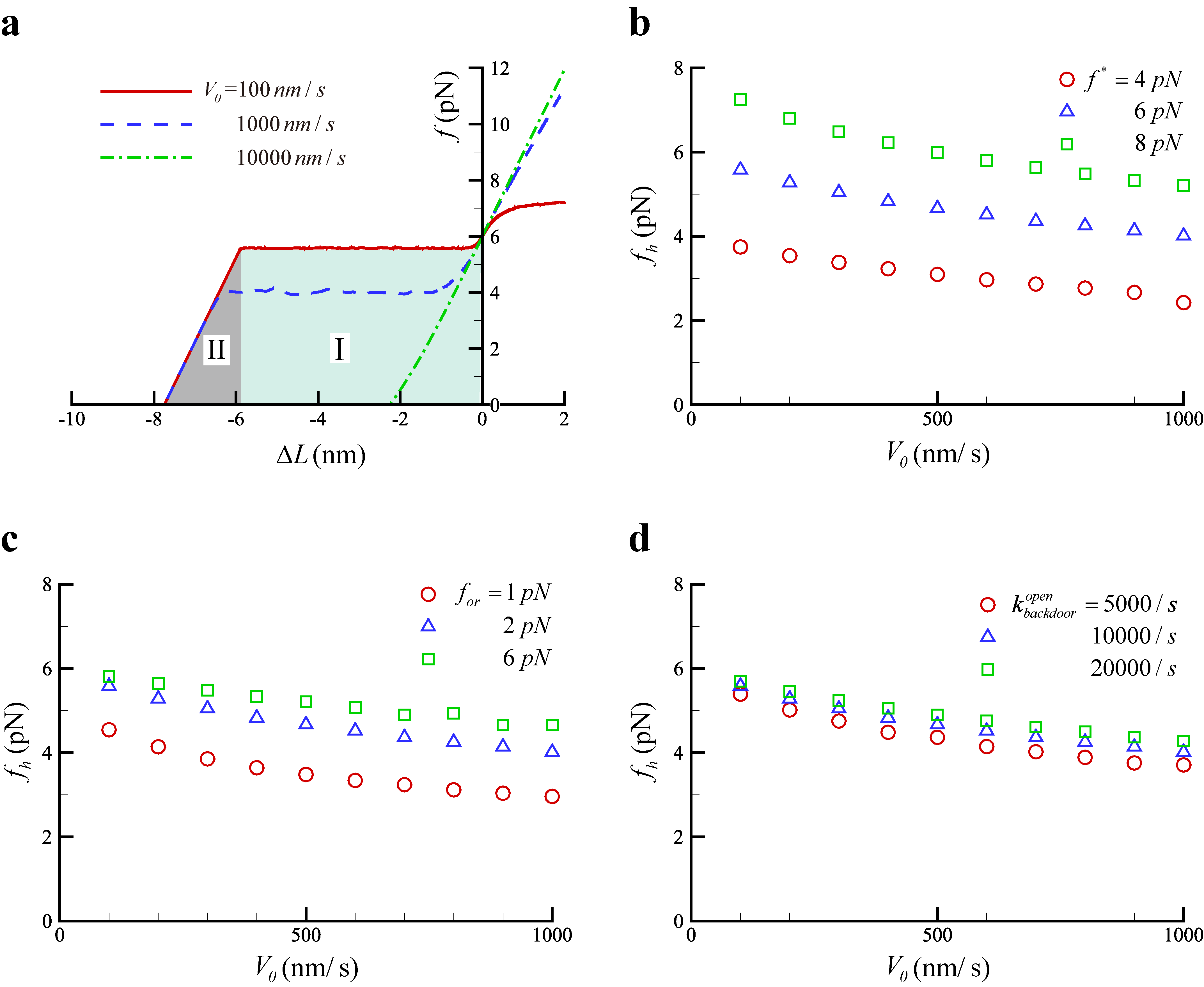


Fig. S1
